# Appropriate training and retention of community doctors in rural areas: a case study from Mali

**DOI:** 10.1186/1478-4491-6-25

**Published:** 2008-11-18

**Authors:** Monique Van Dormael, Sylvie Dugas, Yacouba Kone, Seydou Coulibaly, Mansour Sy, Bruno Marchal, Dominique Desplats

**Affiliations:** 1 Institute of Tropical Medicine, Public Health Department, 155 Nationalestraat, 2000 Antwerp, Belgium; 2 Direction Départementale des Affaires Sanitaires et Sociales, 2 boulevard Murat, BP 3840, 53030 Laval cédex 9, France; 3 Santé Sud, BPE686, Bamako, Mali; 4 Santé Sud, 200 Boulevard National, Le Gyptis, Batiment N, 13003 Marseille, France

## Abstract

**Background:**

While attraction of doctors to rural settings is increasing in Mali, there is concern for their retention. An orientation course for young practicing rural doctors was set up in 2003 by a professional association and a NGO. The underlying assumption was that rurally relevant training would strengthen doctors' competences and self-confidence, improve job satisfaction, and consequently contribute to retention.

**Methods:**

Programme evaluation distinguished trainees' opinions, competences and behaviour. Data were collected through participant observation, group discussions, satisfaction questionnaires, a monitoring tool of learning progress, and follow up visits. Retention was assessed for all 65 trainees between 2003 and 2007.

**Results and discussion:**

The programme consisted of four classroom modules – clinical skills, community health, practice management and communication skills – and a practicum supervised by an experienced rural doctor. Out of the 65 trained doctors between 2003 and 2007, 55 were still engaged in rural practice end of 2007, suggesting high retention for the Malian context. Participants viewed the training as crucial to face technical and social problems related to rural practice. Discussing professional experience with senior rural doctors contributed to socialisation to novel professional roles. Mechanisms underlying training effects on retention include increased self confidence, self esteem as rural doctor, and sense of belonging to a professional group sharing a common professional identity. Retention can however not be attributed solely to the training intervention, as rural doctors benefit from other incentives and support mechanisms (follow up visits, continuing training, mentoring...) affecting job satisfaction.

**Conclusion:**

Training increasing self confidence and self esteem of rural practitioners may contribute to retention of skilled professionals in rural areas. While reorientations of curricula in training institutions are necessary, other types of professional support are needed. This experience suggests that professional associations dedicated to strengthening quality of care can contribute significantly to rural practitioners' morale.

## Background

Staffing of health centres in rural and remote areas is a problem all over the world, affecting particularly sub-Saharan African countries [1-3]. In Mali, a country with critical shortage of health professionals [4], overall availability of skilled health workers is improving, but urban/rural disparities remain strong [5], and staff turnover is high [6,7]. Most health centres in Mali are headed by a nurse, though medical doctors increasingly engage in first line practice, including in rural and remote areas [8-10]. While attraction of rural doctors is steadily rising, there is concern about their long-term retention. In response, an orientation course for recently established rural doctors was set up in 2003, based on a training needs assessment. This paper draws lessons from this experience, focusing on processes and mechanisms operating in the relation between training and retention in rural practice.

### Determinants of staff turnover in rural and remote areas

It is widely recognised that qualified staff turnover tends to be more acute in rural and remote areas than in urban settings [11-13]. This may affect quality of service provision, as new comers need time to establish a relationship with the community. High turnover also affects health service efficiency and team productivity during the initiation period of the newcomer [11]. Turnover may also result in prolonged periods of understaffing of health facilities.

While there is considerable overlap between factors affecting attraction and retention in rural areas, attraction is based on expectations while retention is based on actual working and living experience [14]. Turnover is partially a result of job dissatisfaction, which induces not only de-motivation and absenteeism, but also intentions to quit [11,15].

Job satisfaction, and consequently willingness to remain in a rural post, is influenced by a complex interplay between individual factors, living environment and working conditions [2,16]. Individual professionals have different backgrounds and expectations, but also vary in terms of self-confidence, which impinge on job satisfaction: people with higher self confidence are more likely to persist longer at the task in the face of obstacles than people with lower self-confidence [16].

Living environment has strong influence on job satisfaction in rural areas: inadequate housing, lack of schools and lack of recreational facilities are push factors inducing staff to leave [2,17]. The quality of relations with patients and recognition by local community are all the more essential in rural practice since opportunities to develop alternative social networks are limited [18].

Last but not least, working conditions are major determinants of job satisfaction, According to Herzberg's motivational theory [19], factors that make people dissatisfied at work are different from those motivating them to do a good job. Dissatisfiers relate to working conditions rather than the task itself: low salary, poor career perspectives and training opportunities, unsatisfactory access to supplies and support mechanisms, and disappointing human interactions with colleagues and managers all contribute to a sense of dissatisfaction. In contrast to these extrinsic motivational factors, intrinsic motivation relates to the actual content of work, feelings of achievement, self esteem and self confidence; they contribute to job satisfaction and stimulate performance. According to Herzberg, limiting dissatisfiers motivates a worker to stay, but not to perform better. In line with this theory, some authors argue that avoiding dissatisfiers is more important to promote retention than building particularly high levels of job satisfaction [20]. Others however challenge this view, especially for professionals, and suggest that turnover results as much from low intrinsic job satisfaction than from experiencing difficult working environments [21].

### The Malian Rural Doctors Movement

In 2007, 99 rural doctors were serving in over 13% of Mali's rural community health centres [8]. Explanations of this attraction of doctors into rural first line practice – unusual in Sub Saharan Africa – lie in Mali's health sector reform and health labour market evolution, as well as in an incentive package easing recruitment.

A peculiarity of Malian health sector reform in the 1990s was the development of a network of community health centres managed by locally elected community health associations, in charge of contracting and paying staff. Over the years, community demand for doctors increased. The allocation of Heavily Indebted Poor Countries (HIPC) funds to recruit health staff for rural and remote health areas reduced the financial burden on communities and has accelerated the process since 2001.

In the meantime, the production of medical doctors rose dramatically, from 50 graduates a year in 1998 to 350 in 2006 [22]. Job opportunities for young doctors in Mali, however, have not increased proportionally: public sector recruitment remains restricted, the private sector in Bamako is reaching saturation, opportunities for specialisation are scarce, international brain drain is negligible, and NGOs recruit experienced doctors.

But rural practice is not necessarily a default choice, as teachers from the Faculty of Medicine encourage students to settle in remote areas. Rural practice is also promoted through a package of non-financial incentives provided by an NGO (Santé Sud) and the Malian Rural Doctors Association (Association des Médecins de Campagne): young doctors settling in rural areas usually benefit from interventions aiming at improving living conditions (water, solar panels, motorbike) and working conditions (basic equipment, continuous education, peer support and mentoring). There also is a consensus that financial prospects in rural practice are quite acceptable, except in areas with very low population densities. Indeed, rural doctors are usually paid a basic salary, complemented with bonuses proportional to their workload.

Retention of newly recruited community doctors is however not automatic. Anecdotal observation suggests that they face unforeseen situations for which they feel ill prepared, leading sometimes to early dropout [23]. In response, the NGO and the Rural Doctors Association decided to set up an orientation course for recently established rural doctors. The underlying assumption was that training meeting rural practitioners' needs would strengthen young doctors' technical competences and self-confidence, and consequently contribute to retention.

In this paper, we present the findings of an evaluation of this training course, which addressed the following research questions: (1) What are unmet training needs for rural general practitioners in Mali, (2) What were effects of the orientation course on trainees, and (3) Did the course affect retention, and if so, how?

## Methods

The design and implementation of the training programme was conceived as a participatory action research process, aiming at finding solutions to a practical problem, while generating knowledge to share with a wider audience [24,25]. Unmet training needs were assessed through group discussions with senior rural practitioners, exploring their own difficulties in fulfilling different functions of a rural doctor: clinician, public health practitioner in charge of a community, health centre manager, and – a cross-cutting function – communicator [23]. On this basis, a training programme was designed, consisting of class modules combined with a practicum in a rural health centre run by a senior doctor. Senior doctors involved in the training were appointed by the professional association; selection criteria included peer recognition for excellence, at least 4 years experience in a wide scope of clinical and community-based activities, and good integration in their community and local health system.

In order to assess the effects of the course, we used Kirkpatrick's model of evaluation [26] distinguishing four levels:

• level 1 assesses trainees' reactions, satisfaction and perceived relevance to their work

• level 2 assesses trainees' learning and changes in knowledge, skills and attitudes

• level 3 assesses whether the training changed actual behaviour on the job

• level 4 assesses overall results in terms of production and performance.

This evaluation focused on the three first levels. Reactions (level 1) were assessed by open ended satisfaction questionnaires, eliciting participants' appraisals about the training and its relevance to practice. This was complemented by participant observation and recording of comments during the training sessions, aiming at validating and refining the initial training needs assessment. In order to assess level 2, a reference tool was designed for the practicum, enabling the trainee and his supervisor to define priority learning objectives and assess learning progress; for evaluation of classroom modules, no systematic tests were conducted before and after the sessions, but role plays and supervised exercises were used to monitor learning progress. Finally, effects on actual practice (level 3) were assessed by the NGO coordinators through two follow up visits to each trainee within the year following the training. These visits were primarily meant as supportive supervisions, but also contributed to identify to what extent training contents were implemented in practice; an indicative checklist was designed, including indicators related to quality and affordability of clinical care, community health activities, practice management, team leadership, and interactions with district authorities and hospital.

Finally, we measured retention by assessing the professional career of all doctors who participated in the training from 2003 to 2007. Baseline data of retention of rural doctors prior to the introduction of the programme could, however, not be retrieved.

## Results

### Training needs and programme design

Analysis of the training needs assessment resulted in three main categories: (1) skills and competencies poorly addressed during medical training, including health-centre management, community health programmes, and communication and conflict management; (2) specific clinical skills and knowledge adapted to remote and isolated practice conditions; (3) socialisation to the rural doctor's professional role, and internalisation of norms, values, and practical attitudes characterising rural practice.

On this basis, a yearly training session was organised, starting in 2003, for 10 to 16 recently installed rural doctors, comprising four weeks classroom teaching and four weeks practicum in a health centre run by an experienced rural doctor. The classroom modules addressed clinical skills, health-centre management skills, public health, and communication skills (see Table 1). To stimulate reflection on experience rather than mere knowledge transmission, an interactive pedagogic approach was applied. Each module was prepared and conducted jointly by a "topic expert" (with academic, MOH or NGO background) and a "profession expert" (an experienced community doctor). The involvement of senior doctors in the implementation of modules aimed at taking advantage of their field experience and at providing role models. It was also meant to ensure alignment between classroom sessions and practicum. The four weeks of classroom sessions ended with a global evaluation and a final assembly where experienced community doctors offered final recommendations to the trainees. Skills learned during the modules were implemented during the practicum, under supervision of a senior doctor, individual priority needs being defined jointly by the trainee and his supervisor.

**Table 1:** Objectives and contents of the four training modules

**Clinical skills**: the purpose was not to update clinical competencies, but rather to strengthen decision making capacity in remote contexts, characterised by limited equipment and referral opportunities; the module meant also to address continuity of care and patient-centeredness as characteristics of first line care.
**Practice management**: the module aimed at making participants acquainted with Malian laws and regulations related to health centre functioning, and with practical skills and tools related to financial management, human resource management, and drug management at health centre level.
**Public health**: the module aimed at strengthening abilities to deal with community health issues by articulating curative, preventive and promotional care, using the existing information system for self-evaluation and local planning, establishing dialogue with the community; a second objective was to increase participants' awareness of the role of first line and its relations with other actors within the Malian health care system.
**Communication skills**: the objective was to improve rural doctors' communication skills by making them reflect on their own communication style, and by increasing their awareness of the gaps between their own views and the views of the different actors with whom they interact: patients, communities and their representatives, staff members, local authorities, district health authorities... Topics included health and health seeking behaviour, patient-doctor communication, health education and teamwork

### Assessment of the training programme

Trainees' satisfaction and perceived relevance of the course for the problems they encountered in practice (Kirkpatrick's level 1) was high.

Participants expressed at the beginning of the modules a lack of self-confidence, exacerbated by their social and professional isolation. Not only did they feel unprepared to carry out their clinical duties with limited technical equipment and referral opportunities, but most had not anticipated the cultural gap they experienced when joining their rural post. They reported frequent relational problems detrimental to their social integration: conflicts with the health centre committee (their employer) about working conditions and financial management issues, leadership conflicts with other staff members, absenteeism and misbehaviour of staff, tense coexistence with traditional practitioners, or disagreements with the district medical officer concerning boundaries between first line care and hospital care. They also wanted advice on how to develop trust relationships with the community, and expressed strong feelings of powerlessness in changing health behaviours they considered harmful.

Both the questionnaire responses and the final evaluation session indicated a high level of satisfaction regarding the training. Though they expressed a high demand for further training, in particular in clinical issues, participants described the course as crucial for increasing their self-confidence. A few explicitly stated that it had convinced them to continue practicing in rural areas. Trainees appreciated the interactive pedagogic approach inviting them to reflect on their own experience. They also welcomed the technical inputs, some of which were totally new to most trainees (for instance practice management, or interpretation of routine indicators for self evaluation). Besides technical inputs, young doctors unanimously valued sharing and discussing professional experience with elder practitioners. They were eager to discuss ways of thinking and behaving as a rural community doctor, and senior doctors were eager to share experience. Many problem issues raised during these discussions were related to human relations with the community and at workplace: finding a balance between distance and proximity with community members, acknowledging social hierarchies while remaining equitable, controlling staff behaviour without crystallizing antagonisms from staff and/or community members. Such problems were exacerbated in rural areas, where any work related dispute easily becomes a broader community dispute affecting doctors' social integration, as many staff members are from surrounding community. The final session of recommendations by experienced doctors, illustrated in table 2, provided further opportunities to reflect on professional ethos and to strengthen feelings of shared professional identity and belonging to a group. Senior doctors contributed substantially to an accelerated professional socialisation process, which proved to be a more important training need than anticipated. It should also be noted that several senior doctors declared that, if they had had access to such a training, this would have prevented them from making errors when they first started work.

**Table 2:** Examples of recommendations by senior community doctors to young trainees

• « Avoid favouritism. The chief of the village should queue like any other villager »
• « Never tell a patient that what he is doing is wrong, he wouldn't come back »
• « Respect customs, don't say they are harmful, rather explain that there are other methods »
• « Remain neutral, never take sides with one group of the community against another »
• « Recognise staff members' achievements, and punish misbehaviour: making regulations explicit protects staff members from social pressure from their relatives »
• « Don't monopolise the floor during meetings with the health centre committee; listen to them and give them the opportunity to explain their views »
• « Being a rural doctor requires courage »

While there is strong evidence about participants' satisfaction and perceived relevance of the process and content of training, results related to changes in knowledge and skills (Kirkpatrick level 2) are less conclusive: the tool meant to evaluate learning during practicums was felt as too demanding and was not systematically applied. Informal feed back from teachers and supervisors suggests that skills were acquired, but more detailed information is missing.

Finally, follow up visits by Santé-Sud NGO coordinators confirmed increased self confidence of trainees. It was difficult to assess the extent of actual behavioural changes (Kirkpatrick Level 3) as no baseline data were available. Supervisors' observations suggest overall good levels of team leadership and interactions with district authorities, but persisting difficulties, for part of the trainees, in mastering practice management, developing strategic plans and implementing community based health promotion activities.

### Retention of trained doctors

Between 2003 and 2007, 65 newly installed rural doctors, deployed in all regions of the country, participated in the training. Table 3 shows yearly cohorts and retention in rural practice over the years. At the end of 2007, 55 out of the 65 trained young doctors (85%) were still engaged in rural practice. A few had moved to another rural health centre. The timeframe regarding retention is rather short, as about half of the trainees had less than two years practice at the end of 2007. When focusing on the three first cohorts trained in the period 2003 to 2005, for which we have longer retrospective data, respectively 50%, 77% and 86% were still in rural practice 4, 3 and 2 years after the training. Eight out of 32 trainees for this period were no longer in rural practice end of 2007; five of them left within the two first years of installation. The 8 "dropouts" went for specialist training, got involved in a private practice in the capital city Bamako, or were hired by an NGO.

**Table 3:** Number of newly installed trained doctors and retention in rural practice (2003–2007)

	Number of newly installed doctors trained	Number of doctors still in rural practice end 2007	Number of early dropouts (less than 2 years rural practice)
2003	8	4 (50%)	1
2004	9	7 (77%)	0
2005	15	13 (86%)	2
2006	16	13 (81%)	3
2007	17	17 (100%)	-
Total	65	55 (85%)	5

Source: Santé Sud

## Discussion

Our hypothesis was that appropriate training would strengthen young doctors' competence and self-confidence, and consequently contribute to retention.

Though we lack baseline data, our study suggests that retention of trained rural doctors is relatively high for the Malian context. In 2005, only 21% of the heads of health centres in Mali had been in post for 3 years or more, 29% between 2 and 3 years, and 49% less than 2 years. (Ronse, I. personal communication based on PRODESS monitoring tools, 17/2/2008); these data include urban health centres, where turnover is usually lower [11,12]. Mahe et al [27] found that half of the health workers of 20 Malian health centres trained in 2001 had been replaced by new health workers after 18 months. Similarly, a study conducted in 1997 in 41 community health centres showed that only 29% of the heads of health centres had been working in their settings for more than 2 years [28]. By contrast, our three first cohorts of trainees showed 50% retention after 4 years, 77% after 3 years, and 86% after 2 years.

We cannot conclude, however, that training was the main determinant of retention. First, incentives related to living and working conditions, which influenced rural doctors' attraction, also contribute to retention. Second, other support mechanisms known to foster retention [2,15,29] are provided: mentoring, supervision, and access to further rurally relevant continuous training sessions. While complementary bundles of interventions indeed work better than isolated interventions [30], it is difficult to disentangle their effects.

Our initial hypothesis was that training would increase feelings of being able to do the job. Data concerning reactions of participants (level 1) confirm increased self confidence and high satisfaction with the programme, and suggest that it was indeed appropriately responding to training needs. But methodological weaknesses in assessing changes in competence (level 2) and performance (level 3), prevent us from concluding that self confidence results indeed from superior skills and knowledge. On the basis of follow up visits, we may however assume that the training raised awareness about different roles of rural practitioners, and provided information and technical tools useful for carrying out their novel functions. Part of the training dealt with relatively simple practical issues likely to affect working conditions and job satisfaction: good drug management avoids shortages and subsequent frustrations; tools for transparent financial management prevent conflicts with health centre committee; clarification of regulations limits misunderstandings with public authorities.

Participant observation of the experience allows us to hypothesise two further mechanisms operating in the relation between this continuous training experience and retention.

First, the course not only addressed knowledge and skills, but also professional socialisation, i.e. "the learning of attitudes, norms, self images, values, beliefs and behavioural patterns" associated with professional practice [31]. Group discussions contributed to internalisation of ways of thinking, feeling and acting as a rural doctor. The crystallisation of rural doctors' professional identity during this process fostered self-esteem: rural practice was portrayed as a demanding profession of high added social value, but also a rewarding profession, as rural doctors provide a wide range of comprehensive care and have a high level of autonomy. This socialisation process resulted from the method as much as the content of training, addressing a homogeneous group of rural doctors, involving senior doctors as role models, and emphasising group work and experience sharing.

Second, appropriate continuous training for rural practice contributes to retention also by alleviating feelings of professional isolation [32]. The training process of Malian rural doctors had a strong supporting function, strengthening their sense of belonging to a group and their ability to resist social and professional isolation. It should be noted that the course turned out to be an intense moment in the professional association's life, contributing to the establishment of lasting supportive relationships with peers and mentors, which probably enhanced job satisfaction.

The experience highlights the importance of intrinsic satisfaction for retention: improved retention was not only the consequence of diminishing dissatisfiers, it also resulted from self esteem as a rural practitioner, helping to resist dissatisfiers. In this project, clarifying a sense of mission and professional ethos was facilitated by a professional association. A mission oriented organisational culture initiated through other means could achieve similar effects on retention.

The underlying mechanisms – i.e. increasing self confidence in own skills, self esteem as rural doctor, and sense of solidarity within a group sharing a common professional identity – need to be framed in the specific Malian context. Indeed, the training course had the effects discussed above, because the graduates of medical school are not enabled to fully function in posts at the first line in rural areas. This type of course, therefore, will be more effective in settings where medical education is oriented towards hospital or district-level medicine, and where rural practice entails novel professional roles for medical doctors [33].

## Policy implications

As the training consists to a large extent in compensating for shortcomings of the medical school-based training, a reasonable approach would be to incorporate this kind of training in the basic curriculum. Though highly desirable, this is but a first step. Medical school can, and should, address training needs pertaining to clinical skills, rational use of technical procedures, community health, practice management and communication, which are indeed useful in any situation. Socialisation to rural practice could also be developed through specific rural practice programmes.

However, once installed, rural doctors face challenging situations and are in need for peer-based reflection and support, especially when they start with rural practice and are at highest risk of encountering discouraging critical incidents. Even if at academic level, the medical curriculum was revised to improve alignment with practice, there would still be a need for structured professional support. In the project discussed here, this is ensured through a package consisting of continuous training, mentoring, supportive supervision and regular meetings, all provided within a professional association with support from an NGO. Such a package, more than the training alone, is likely to promote retention, at least during a few years.

## Conclusion

While incentive packages condition acceptance to work in rural and remote areas, self confidence and self esteem affect decisions to remain in these posts. Appropriate training can contribute to retention by improving skills, changing attitudes and enhancing self confidence. Other support mechanisms are however necessary to help practitioners to cope with social and professional isolation. The Malian Rural Doctors experience suggests that a professional association, dedicated to strengthening quality of care, can contribute significantly to rural practitioners' morale.

## Competing interests

The authors declare that they have no competing interests.

## Authors' contributions

MVD and SD both contributed to the design, implementation and evaluation of the programme and drafted the manuscript. YK carried out the preliminary analysis of training needs, contributed to the design, implementation and evaluation of the programme and revised the manuscript. SC and MS contributed to the design, implementation and evaluation of the programme, collected follow up information about trainees and revised the manuscript. BM drafted and revised the manuscript. DD contributed to the design and evaluation of the programme and revised the manuscript.
